# Unravelling the Association Between Trait Mindfulness and Problematic Social Media Use in Youth

**DOI:** 10.3390/ijerph22101479

**Published:** 2025-09-25

**Authors:** Elisa Galli, Marta Sannino, Zidane Dridi, Marco Giancola

**Affiliations:** 1 Department of Neuroscience, Imaging and Clinical Sciences, University G. D’Annunzio of Chieti-Pescara, 66100 Chieti, Italy; elisa.galli@phd.unich.it; 2 Department of Biotechnological and Applied Clinical Sciences, University of L’Aquila, 67100 L’Aquila, Italy; marta.sannino@graduate.univaq.it (M.S.); zidane.dridi@student.univaq.it (Z.D.)

**Keywords:** addiction, technology, mindfulness, personality, regression, social media, youth

## Abstract

The compulsive and unregulated use of social media, also known as problematic social media use (PSMU), has raised increasing concerns about its detrimental impact on psychological well-being and daily functioning among young individuals. Understanding the psychological mechanisms underlying this behavioural pattern is essential, with personality traits emerging as a particularly relevant area of investigation. While some personality traits, such as narcissism, have received substantial attention, others remain overlooked. Through a cross-sectional design, this study examined how trait mindfulness, as captured in terms of awareness and acceptance, predicts PSMU while accounting for sociodemographics (age, sex, and education) and both grandiose and vulnerable narcissism. A total of 180 participants (mean_age_ = 22.16 years; SD_age_ = 2.47 years; 95 females) completed the Narcissistic Personality Inventory-13, the Hypersensitive Narcissism Scale, the Philadelphia Mindfulness Scale, and the Bergen Social Media Addiction Scale. Regression analyses indicated that only the acceptance of trait mindfulness significantly predicted PSMU. These findings suggest that cultivating a non-judgmental and open stance towards internal experiences may protect against PSMU through affect regulation and emotional acceptance.

## 1. Introduction

In the contemporary digital era, characterised by the rapid proliferation of Internet technologies and the widespread availability of electronic devices, social media platforms have become deeply embedded in individual daily life [1,2]. Over recent years, the global number of social media users has increased exponentially, particularly among adolescents and young adults [3,4,5]. While these platforms, such as Instagram, Facebook, X, and TikTok, offer valuable opportunities for information sharing and social interaction, their use is not without risks. In particular, excessive and unregulated engagement can manifest as Problematic Social Media Use (PSMU) [6,7,8,9].

PSMU is a complex phenomenon that refers to compulsive, maladaptive, and dysregulated use of social media [10,11,12,13,14]. As shown in previous research, it relies on six core symptoms, such as *salience* (excessive preoccupation for social media use and neglect of everything else), *mood modification* (consistent use of social media to alter mood), *tolerance* (increasing social media use over time to achieve the same initial mood modifications), *withdrawal effects* (distressing emotional and physical symptoms associated with restricted social media use), *conflict* (psychological and interpersonal difficulties arising from excessive social media use, including deteriorated social relationships, compromised employment, and/or education), and *relapse* (a swift return to problematic social media use, after a period of abstinence) [15,16,17].

Although the detrimental effects of PSMU on health and well-being have gained growing scientific interest, the main psychological factors that may contribute to its onset, escalation, and persistence over time remain overlooked [18,19,20].

Given this gap, the present study investigates the role of trait mindfulness in PSMU, after controlling for sociodemographics (age, sex, and education) and personality traits widely associated with PSMU, such as grandiose and vulnerable narcissism.

### 1.1. Literature Review

The Social Online Self-Regulation Theory (SOS-T) [21] proposes that individuals actively engage with social media platforms to regulate self-related processes, including self-esteem, identity coherence, and affective states. Social media environments offer asynchronous communication, which allows time to process and craft responses; strategic self-presentation, which enables the management of one’s self-image; and immediate feedback (e.g., likes, comments), which can serve as social reinforcement. These features facilitate self-regulatory mechanisms and help individuals maintain psychological stability. In addition, SOS-T conceptualises social media use as an active, goal-oriented behaviour directed toward constructing, affirming, and sustaining a coherent self-concept rather than as passive consumption.

In parallel, the Compensatory Internet Use Theory (CIUT) [22,23] suggests that individuals frequently turn to digital platforms to cope with offline psychosocial difficulties or emotional distress. This pattern of compensatory use is pervasive among individuals experiencing social anxiety, loneliness, or low self-esteem, who may perceive online interactions as less socially demanding and more controllable than face-to-face communication [23]. The structural features of social media, such as anonymity, asynchronous communication, and algorithmically curated content, can act as buffers against real-world social challenges. However, these same features may also foster dependency, leading to maladaptive patterns including PSMU [23]. The CIUT further highlights the instrumental role of social media in emotion regulation, functioning as a distraction from intrusive or distressing thoughts and as a temporary source of belonging and validation [22].

Taken together, SOS-T and CIUT suggest that PSMU may arise when individuals attempt to manage self-related concerns and regulate affective states in ways that, over time, become maladaptive. Within this framework, narcissism has emerged as a prominent personality factor linked to PSMU [6,24,25]. Narcissism is characterised by a need for admiration, beliefs in uniqueness, and an inflated self-evaluation, which may coexist with underlying insecurity [26]. It comprises two main facets: grandiose narcissism, marked by high sociability, assertiveness, and exhibitionism, and vulnerable narcissism, marked by avoidance, chronic shyness, low self-esteem, withdrawal, and negative emotionality [27,28,29,30].

Although both grandiose and vulnerable narcissism are associated with PSMU, they appear to relate to it through different social strategies aimed at gaining attention and admiration [31,32,33]. Social media platforms can provide an ideal environment for both types: for grandiose narcissists, they offer opportunities for self-promotion, social dominance, and the public reinforcement of self-image; for vulnerable narcissists, they provide a less threatening context for social interaction and a means of obtaining emotional reassurance [6,34]. Consistent with SOS-T, grandiose narcissists may use social media to enhance their image and consolidate a dominant self-presentation. In contrast, vulnerable narcissists may use these platforms as a way to manage social anxiety and bolster unstable self-esteem [6,25]. Evidence suggests that grandiose narcissism is positively associated with PSMU, partly through the gratification derived from likes, comments, and other forms of visible admiration [34,35,36,37,38]. Conversely, vulnerable narcissism is linked to stronger preferences for online over offline interactions and to compulsive use patterns, often as a means of mood regulation or avoidance of real-world social demands [39,40,41]. From a CIUT perspective, the insecurities, low self-esteem, and fear of rejection characteristic of vulnerable narcissism predispose individuals to use social media as a means of reassurance and validation [42,43,44], while individuals high in grandiose narcissism may similarly compensate for their need for admiration and exhibitionism through continuous online validation [35,39].

Despite the strong empirical support for narcissism as a risk factor for PSMU, other personality traits that may shape online behaviour remain underexplored. Of particular interest is trait mindfulness, a dispositional characteristic associated with heightened self-awareness, adaptive emotion regulation, and reduced engagement in maladaptive coping behaviours [45,46,47]. Trait mindfulness is defined as the tendency to attend to present-moment experiences intentionally and non-judgmentally [48,49,50]. It is generally conceptualised in two dimensions: awareness, referring to the capacity to perceive and attend to internal and external stimuli; and acceptance, referring to the ability to observe one’s thoughts and feelings openly, without avoidance or judgment [51]. Higher levels of trait mindfulness, especially acceptance, have been linked to lower anxiety, reduced rumination and impulsivity, and enhanced emotional control, all of which may reduce the likelihood of engaging in maladaptive digital behaviours such as PSMU [1,14,52].

The relationship between mindfulness and PSMU can also be understood through the lens of SOS-T and CIUT. Both theories imply that individuals often turn to social media as a form of self-regulation when experiencing psychological distress or unmet needs. From this standpoint, low mindful awareness and, particularly, deficient acceptance may increase vulnerability to PSMU by impairing emotion regulation and promoting avoidance-based coping. Conversely, high trait mindfulness may enable individuals to tolerate unpleasant internal states without resorting to compulsive online behaviours. By breaking the self-reinforcing compensatory cycle that sustains PSMU, trait mindfulness, particularly acceptance, could serve as a protective factor that not only reduces the frequency of maladaptive social media use but also promotes more adaptive self-regulatory strategies [6,53].

### 1.2. The Present Study

Building on the SOS-T and the CIUT, the present study addresses a critical gap in the literature: the role of trait mindfulness in PSMU. While previous research has linked certain personality traits particularly grandiose and vulnerable narcissism to PSMU, the protective potential of trait mindfulness and specifically its distinct components, namely awareness and acceptance, have not been systematically examined within these theoretical frameworks.

The SOS-T conceptualises social media use as a strategy to regulate self-related processes, whereas CIUT frames it as a means of compensating for offline difficulties and emotional distress. Both suggest that deficits in self-regulation increase susceptibility to maladaptive online behaviours. Within this context, awareness—sustained attention to present-moment experience—may help users monitor and control their online activity, interrupting compulsive patterns. Acceptance—the non-judgmental acknowledgment of internal states—may reduce emotional avoidance, decreasing reliance on social media for mood regulation and reassurance.

By integrating these perspectives, this study proposes that higher levels of awareness and acceptance weaken the self-reinforcing cycles that maintain PSMU. It was therefore hypothesised that awareness and acceptance facets of trait mindfulness are negatively associated with PSMU, even after controlling for sociodemographics (age, sex, and education) and established personality predictors of PSMU, such as grandiose and vulnerable narcissism.

## 2. Materials and Methods

### 2.1. Study Design

This study employed a cross-sectional, correlational design to investigate the relationship between trait mindfulness and PSMU, while controlling for grandiose and vulnerable narcissism. In order to control for potential spurious effects of sociodemographics, age, gender, and education were also included in the analysis. All measures were administered online via an online platform and data collection occurred between January and July 2023.

The study adhered to the ethical standards of the Declaration of Helsinki and was approved by the Institutional Review Board of the University of L’Aquila (protocol number: 119943).

### 2.2. Participants and Procedure

An a priori power analysis was conducted using the G*Power software (version 3.1.9.7) [54] to determine the minimum sample size for a multiple regression analysis: test family: “*F* test analysis”; statistical test: “Linear multiple regression: fixed model, *R*^2^ deviation from zero”; type of analysis: “A priori: Compute required sample size—given *α*, power and effect size”, *α* err prob = 0.05, power (1-*β* err prob) = 0.95, mean effect size *f*^2^ = 0.15 (medium effect), and a maximum number of predictors = 7. The recommended minimum sample size was *n* = 153.

A total of 210 participants took part in the study. After removing missing (*n* = 22) and outliers (*n* = 8), the final sample consisted of 180 individuals (mean_age_ = 22.16 years; SD_age_ = 2.47 years; 95 females). Regarding educational background, participants have shown an average of 14.63 years of education (SD = 1.55 years) with a range between 13 and 18 years. Furthermore, no participants have declared previous experience or exposure to mindfulness training. All participants have confirmed the absence of any neuropsychological or psychiatric conditions that could potentially influence the results of the study and have indicated regular engagement with social media platforms. Table 1 reports all socio-demographic features of the research sample.

Participants were recruited through online advertisements on social media platforms (Facebook, Instagram, and WhatsApp) as well as via word-of-mouth. Before engaging in the survey, participants were informed about the study’s aim through an online informed consent page. The survey contained a first part about socio-demographics and individuals’ experiences in mindfulness, followed by a second part in which participants completed self-report questionnaires about grandiose and vulnerable narcissism, trait mindfulness, and PSMU. No rewards were provided for taking part in the online survey. All data were recorded anonymously, and participants were assured of the protection of their identity and personal information.

### 2.3. Measures

*Socio-demographic questionnaire*. Collected data on age, gender, and level of education (years).

The *Narcissistic Personality Inventory–13* (NPI-13) [55] captures the core features of grandiose narcissism, including entitlement, leadership, and exhibitionism. The NPI-13 comprises 13 items (e.g., *I will usually show off if I get the chance*) along a 7-point Likert scale ranging from 1 (strongly disagree) to 7 (strongly agree). The mean score has been computed, with a high score indicating high levels of grandiose narcissism. In previous studies, this measure demonstrated good psychometric properties [56]. In this research, the internal consistency reliability was Cronbach’s *α* = 0.88.

The *Hypersensitive Narcissism Scale* (HSNS) [57] measures the main features of vulnerable narcissism, such as hypersensitivity to criticism, social withdrawal, and emotional insecurity. The HSNS comprises 10 items (e.g., *I feel that I have enough on my hands without worrying about other people’s trouble*) along a 7-point Likert scale ranging from 1 (very untrue of me) to 7 (very true of me). A total score has been obtained by summing the item responses, with higher scores reflecting greater levels of vulnerable narcissism. Previous research indicated that the HSNS has good psychometric properties [56]. In this research, the internal consistency reliability was Cronbach’s *α* = 0.71.

The *Philadelphia Mindfulness Scale* (PHLMS) [51,58] consists of 20 items assessing the two core components of mindfulness: *awareness* and *acceptance*. The *awareness* subscale measures the individual’s ability to attend to present-moment experiences (e.g., thoughts, emotions, bodily sensations). The *acceptance* subscale assesses the tendency to respond to these experiences in a non-judgmental and open manner. Each subscale contains 10 items rated on a 5-point Likert scale from 1 (never) to 5 (very often), with higher scores indicating greater dispositional mindfulness in the respective domain. The PHLMS demonstrated good psychometric properties [58]. In this study, the internal consistency reliability was Cronbach’s *α* = 0.81 and 0.76 for awareness and acceptance, respectively.

The *Bergen Social Media Addiction Scale* (BSMAS) [59] evaluates PSMU through 6 items (e.g., *how often during the last year have you felt an urge to use social media more and more?*) along a 5-point Likert scale ranging from 1 (very rarely) to 5 (very often). The BSMAS is designed to capture the core symptoms of PSMU based on Griffiths’ model of addiction [16], capturing key dimensions, such as salience, tolerance, mood modification, withdrawal, conflict, and relapse. A total score was computed by summing the item responses, with higher scores indicating more severe PSMU. In previous studies, the BSMAS demonstrated excellent psychometric properties [20]. In this study, the internal consistency reliability was Cronbach’s *α* = 0.90.

## 3. Results

**Figure 1:**
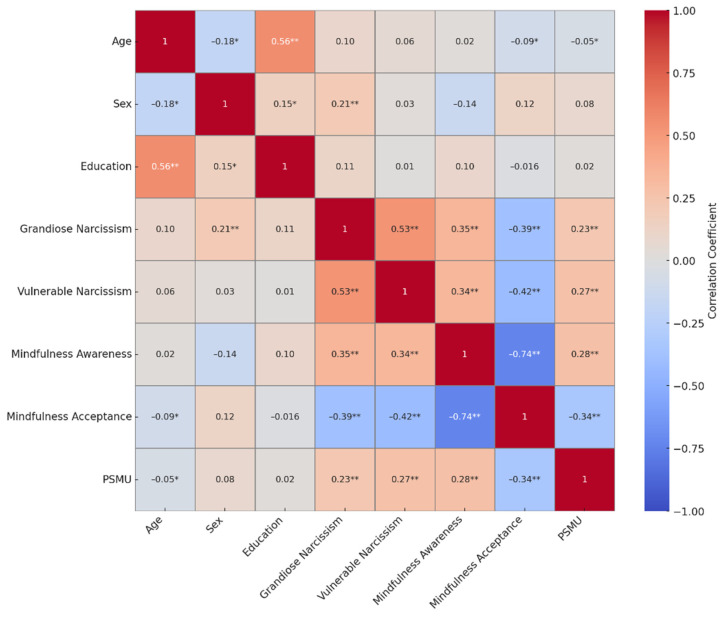
Heatmap of the correlations among all study variables. Note. PSMU = Problematic Social Media Use. *Note.* * *p* < 0.05; ** *p* < 0.01.

A Pearson bivariate correlation analysis (Figure 1) was conducted to examine the associations among all study variables. As shown in Table 1, age was positively associated with mindfulness awareness (*r* = 0.35, *p* < 0.001) and negatively associated with PSMU (*r* = −0.16, *p* < 0.05). Grandiose narcissism and vulnerable narcissism were positively associated with PSMU (*r* = 0.231, *p* < 0.01 and *r* = 0.27, *p* < 0.001, respectively). Importantly, mindfulness acceptance was negatively associated with PSMU (*r* = −0.36, *p* < 0.001).

A three-step hierarchical multiple regression was conducted to examine the predictive value of demographic variables, narcissism traits, and mindfulness components on problematic social media use (PSMU). In Step 1, age, sex, and education were entered, explaining a nonsignificant amount of variance in PSMU (*R*^2^ = 0.01, *p* = 0.610). In Step 2, grandiose and vulnerable narcissism were added, resulting in a significant improvement in model fit (*R*^2^ = 0.09, *p* = 0.001). In the final model (Step 3), the inclusion of mindfulness awareness and acceptance further improved prediction (*R*^2^ = 0.12, *p* = 0.002), bringing the total explained variance to 16%. Only mindfulness acceptance emerged as a significant negative predictor of PSMU (*β* = −0.26, *p* = 0.02). Table 2 summarizes the regression analysis.

## 4. Discussion

The present study examines the association between trait mindfulness (operationalised through awareness and acceptance facets) and PSMU, while controlling for sociodemographics (age, sex, and education) and established personality predictors, such as grandiose and vulnerable narcissism. Results partially confirm the research hypothesis (i.e., awareness and acceptance facets of trait mindfulness are negatively associated with PSMU, even after controlling for established personality predictors of PSMU, such as grandiose and vulnerable narcissism), revealing that only acceptance is negatively associated with PSMU.

The predictive role of acceptance underscores the importance of a non-judgmental, open stance toward internal experiences as well as acknowledging thoughts, emotions, and sensations without suppression or avoidance, and with curiosity and compassion [60]. This attitude appears to buffer against maladaptive coping strategies, such as excessive or compulsive social media use. Acceptance may enable individuals to tolerate distressing affective states without resorting to digital platforms as a means of distraction or emotional escape. These results align with prior evidence linking acceptance to more adaptive emotion regulation, reduced anxiety, and lower levels of rumination [61,62].

From the perspective of the SOS-T and the CIUT, acceptance may directly counteract the self-regulatory deficits that drive PSMU. By reducing avoidance tendencies and fostering emotional openness, acceptance may interrupt the compensatory cycle whereby individuals use social media to manage negative affect or unmet psychological needs. In this sense, acceptance could weaken the reinforcing loop between transient emotional relief and sustained problematic engagement.

In contrast, the absence of a notable effect for awareness may suggest that focusing attention on the present moment, understood as the capacity to consciously attend to experiences as they occur, might not suffice by itself to disrupt unhealthy behaviours, such as PSMU [60]. Even though there exists a positive connection between awareness and PSMU at the bivariate level, this link disappears once additional factors are accounted for. This suggests that awareness, in absence of acceptance, may amplify the fixation on distressing material without alleviating emotional susceptibility. Without a non-judgmental and accepting approach, concentrating on negative internal states could even heighten subjective distress and, in turn, increase the likelihood of problematic behaviour.

By differentiating between awareness and acceptance, this study extends existing theoretical frameworks on PSMU. Within SOS-T, acceptance may enhance self-regulatory capacities by reducing the need to engage in strategic self-presentation or seek validation to stabilise self-esteem. Within CIUT, acceptance may diminish the motivation to use social media as a compensatory mechanism for offline emotional or relational difficulties. These results therefore support a more nuanced integration of mindfulness into these models, highlighting acceptance as the critical facet for reducing reliance on digital platforms for emotion regulation.

From an applied perspective, these findings have direct implications for interventions targeting PSMU. Acceptance-based approaches, such as Acceptance and Commitment Therapy (ACT) and Mindfulness-Based Cognitive Therapy (MBCT), could be leveraged to cultivate emotional tolerance, reduce avoidance, and promote adaptive coping strategies [62,63,64]. Such interventions may be especially effective for younger populations if adapted to developmental needs, incorporating age-appropriate language, experiential exercises, and digital literacy components. Integrating acceptance training into preventive programmes in schools or community settings could strengthen emotional resilience and reduce vulnerability to compulsive social media use.

Several limitations should be noted. First, the cross-sectional design prevents inferences about causal relationships among the study variables. Second, the exclusive use of self-report measures may have introduced biases, including social desirability and inaccuracies in self-perception. Third, the sample’s Western cultural background limits the generalisability of the findings to other cultural contexts [65]. Finally, the study examined only trait mindfulness and narcissism, leaving other potentially relevant psychological factors, such as cognitive styles, impulsivity, emotion regulation, self-esteem, self-presentation concerns, insecurity, and social anxiety [66,67,68,69,70], to be addressed in future research.

## 5. Conclusions

In conclusion, the present findings identify acceptance—a core facet of trait mindfulness—as a protective factor against PSMU. Unlike awareness, acceptance appears to buffer individuals from maladaptive digital coping by fostering a non-judgmental openness to internal experiences, thereby disrupting self-reinforcing cycles of compulsive use. These results refine theoretical models, such as SOS-T and CIUT, by highlighting the distinct contribution of trait mindfulness components in digital behaviour regulation.

## Figures and Tables

**Table 1:** Main socio-demographic features of the research sample.

	Frequency (%)	*M* (*SD*)
*Age*		22.16 (2.47)
*Gender*		
Male	85 (47.2)	
Female	95 (52.8)	
*Education*		
High School	84 (46.7)	
Bachelor’s degree	93 (51.7)	
Master’s degree	3 (1.7)	

**Table 2:** Summary of the Hierarchical Regression Analysis.

	B	SE	β	*t*	*p*
*Step 1*					
Age	−0.17	0.20	−0.07	−0.80	0.42
Sex	0.85	0.87	0.07	0.97	0.34
Education	0.26	0.32	0.07	0.77	0.44
*R*^2^ = 0.01					
*F*(3, 176) = 0.61					
*Step 2*					
Age	−0.23	0.20	−0.10	−1.13	0.26
Sex	0.43	0.87	0.04	0.50	0.62
Education	0.24	0.32	0.07	0.74	0.46
Grandiose Narcissism	0.72	0.56	0.11	1.27	0.21
Vulnerable Narcissism	0.16	0.07	0.21	2.47	0.01
*R*^2^ = 0.09					
*F*(5, 174) = 3.51 **					
*Step 3*					
Age	−0.18	0.20	−0.08	−0.91	0.37
Sex	1.06	0.87	0.09	1.22	0.23
Education	0.09	0.32	0.03	0.29	0.77
Grandiose Narcissism	0.19	0.57	0.03	0.33	0.74
Vulnerable Narcissism	0.10	0.07	0.13	1.51	0.13
Mindfulness Awareness	0.04	0.09	0.05	0.44	0.66
Mindfulness Acceptance	−0.24	0.10	−0.26	−2.37	0.02
*R*^2^ = 0.16					
*F*(7, 172) = 4.50 ***					

*Note.* ** *p* < 0.01; *** *p* < 0.001.

## Data Availability

The dataset will be made available upon request.
